# An emergency case of small bowel obstruction due to multiple gallstones in a limited resource setting

**DOI:** 10.1016/j.ijscr.2019.09.011

**Published:** 2019-09-23

**Authors:** Sumadi Lukman Anwar, Hartolo Pringgo Handoko, Widya Surya Avanti, Lina Choridah

**Affiliations:** a Department of Surgery – Faculty of Medicine, Public Health and Nursing, Universitas Gadjah Mada/Dr Sardjito Hospital, Yogyakarta 55281, Indonesia; b Department of Surgery, Soeradji Tirtonegoro Hospital, Klaten 57424, Indonesia; c Department of Radiology – Faculty of Medicine, Public Health and Nursing, Universitas Gadjah Mada/Dr Sardjito Hospital, Yogyakarta 55281, Indonesia

**Keywords:** CT-scan, computed tomography scan, Gallstone ileus, Emergency surgery, Low-resource setting, Enterolithotomy, Cholecystectomy

## Abstract

**Introduction:**

Gallstone ileus is an uncommon intestinal obstruction caused by impaction of gallstones passing through the cholecysto-intestinal fistula. Diagnosis of gallstone ileus remains a major challenge because the symptoms and signs are unspecific and the recommended laboratory examinations are not always available particularly in limited health resource settings including in Indonesia. In addition, treatment of choice is still debatable involving enterolithotomy only with or without additional cholecystectomy and fistula repair in one or two-stage surgery.

**Presented case:**

A 49 years old overweight Javanese woman presented in the emergency room with nausea, frequent vomiting, abdominal distension, and absence of defecation in the past 3 days before admission. Previous abdominal surgery and history of biliary disease were not reported during anamnesis. Plain abdominal X-rays revealed small bowel obstruction. Upon emergency laparotomy, the mechanical bowel obstruction was caused by multiple gallstones in the terminal ileum.

**Discussion:**

Although the incidence of gallstone ileus is 5% of all intestinal obstruction cases, the mortality rate is around 25%. Diagnosis is often difficult and half of the cases are diagnosed intraoperatively. Surgical management is still controversial involving enterolithotomy with or without cholecystectomy and fistula repair in one or two stage procedure.

**Conclusions:**

Although the incidence is rare, elaborating thorough anamnesis, physical and basic laboratory examinations in gastrointestinal obstruction should consider gallstone ileus as a potential differential diagnosis particularly in a patient with any risk factors of the biliary disease. Enterolithotomy only might be acceptable for any emergency case due to the equivalent clinical outcomes and relatively low complications.

## Highlights

• Gallstone ileus is a rare condition accounting for 5% of all intestinal obstruction cases in which the mortality rate is relatively high (25%).
• Preoperative diagnosis is a major challenge because the symptoms are unspecific and diagnosis is often established intraoperatively.
• Surgical treatment with enterolithotomy alone is recommended for geriatric patients with concomitant comorbidities.
• Enterolithotomy alone might also be suitable in the case of emergency because of less clinical complications and comparable outcomes.

## 1 Introduction

Gastrointestinal obstruction due to gallstones is an infrequent emergency condition caused by impaction of stones derived from the gallbladder passing through the cholecysto-intestinal fistula. Although the incidence is only 5% of all intestinal obstruction, the mortality rates range around 20–25% of total cases that are frequently found in elderly patients with comorbid conditions [[1], [2], [3]]. Because the symptoms and signs are nonspecific, diagnosis of gallstone ileus remains insidious and relatively difficult. At the emergency room, gallstone ileus might present as acute or intermittent episodes of mechanical bowel obstruction accompanied by nausea, vomiting, abdominal distension, and crampy pain. The intensity of symptoms increases due to the advancement of stone impaction. Biliary symptoms including acute cholecystitis and jaundice are found only in 15% of cases [1,4]. Given the large variety of clinical presentation, imaging using X-ray, sonography, and Computed Tomography scan (CT-scan) plays a vital role in the establishment of the diagnosis of gallstone ileus. However, in the areas of the world with limited health facilities including in Indonesia, imaging is not always available especially in the case of emergency presentation. Therefore, upon the unspecific presentation of bowel obstruction with or without biliary symptoms, patients with risk factors of gallstones should be suspected for gallstone ileus. The standard management of gallstone ileus also remains disputed whether enterotomy alone or in combination with cholecystectomy and fistula closure is sufficient to prevent recurrence and further biliary complications [4,5]. In this report, we described a case of a middle age woman with small bowel mechanical obstruction due to gallstone ileus and presented the report in compliance to the SCARE 2018 guidelines [6].

## 2 Case report

**Fig. 1:**
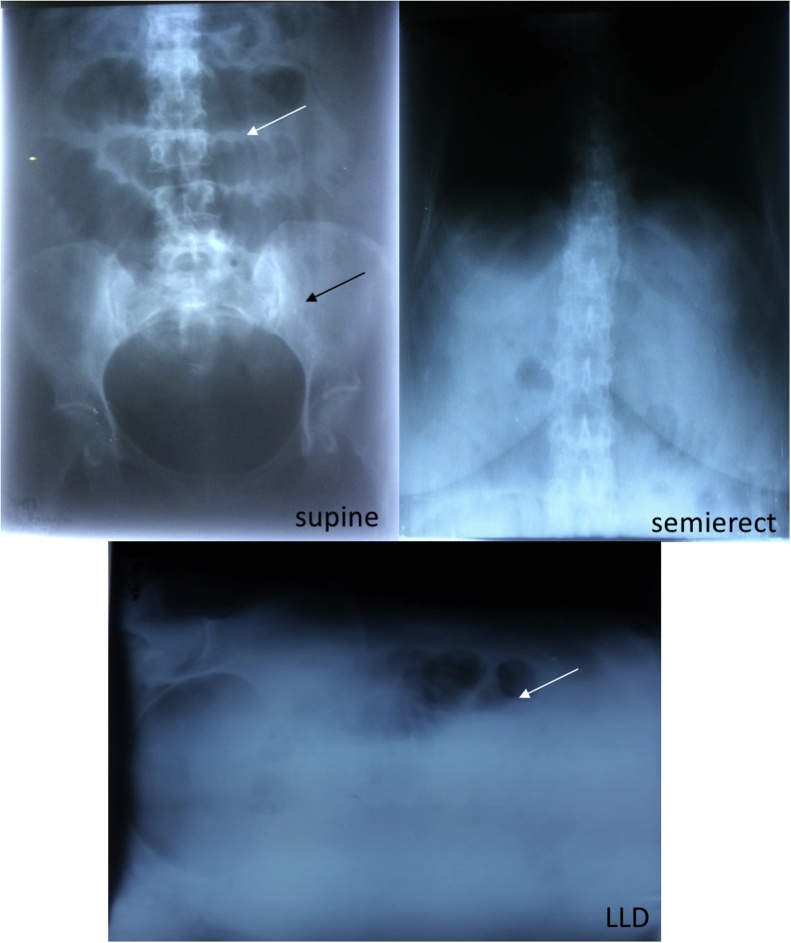
Abdominal X-ray revealed findings of small bowel obstruction. Supine view demonstrated dilatation of multiple loops of small bowel (white arrow). Large bowel was collapsed and absent of air in the rectosigmoid (black arrow). At the lateral left decubitus (LLD), multiple air-fluid levels were shown in the dilated loops of small bowel obstruction (white arrow).

**Fig. 2:**
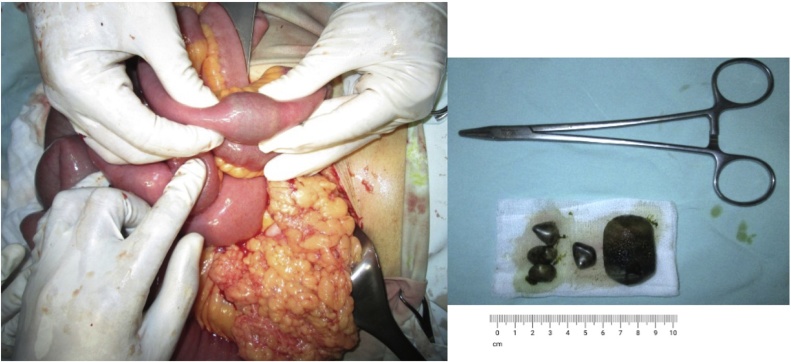
Exploratory findings during surgery in a patients with small bowel obstruction due to gallstone ileus. An impacted gallstone with a size of 4.5 cm × 3.5 cm × 3.2 cm and 4 small gallstones were identified in the distal ileum, 40 cm from the ileocecal junction (right panel). Additional 4 smaller gallstones were found in the proximal part of the ileum (left panel).

We reported the case of a 49 years old Javanese woman with an acute presentation of nausea, vomiting, and abdominal distension at the emergency room of a hospital in the south part of Central Java, Indonesia. She complained about loss of appetite, constipation, and inability to pass gas (flatus). The symptoms intensified during the past 2 days and green vomit appeared at the day of admission. According to anamnesis, there was no history of trauma, previous surgery, liver and biliary problems as well as metabolic diseases. Physical examination revealed tachycardia (112 beats/min), tachypnoeic (22 times/min), non-tender abdomen with hyperactive bowel sounds. Laboratory tests showed leukocytosis (14,970/μL). Abdominal X-ray showed loop dilatation of the small bowel (Fig. 1). Abdominal CT-scan with contrast was available only during daily working hours of the hospital. She was managed with fluid rehydration and antibiotics. The patient was then assigned for an emergency laparotomy. Abdominal exploration revealed massively dilated loops of the entire small bowel to the distal ileum. The obstruction was found 40 cm from the terminal ileum where an antimesenteric enterotomy was performed revealing a large gallstone with the size of 4.5 cm × 3.5 cm × 3.2 cm and 4 small gallstones (1 cm in diameter each) (Fig. 2). After removal of the gallstones, the enterotomy was repaired with seromucous interrupted suturing. The patient was discharged 5 days after the surgery without any postoperative complications and remained symptomatic-free after two year outpatient follow-up.

## 3 Discussion

Patients with gallstone bowel obstruction present mostly in the emergency room with nonspecific clinical symptoms. More than 30% of the patients do not show biliary-related symptoms and mostly present with clinical features of mechanical gastrointestinal obstruction including abdominal pain, distension, and vomiting (bilious or faeculent vomitus in proximal or distal obstruction, respectively) [1]. Although it predominantly affects patients who are elderly [1,7], we reported a case of gallstone ileus in a middle age woman. Overweight was the only risk factor identified in our case. Laboratory tests often show leucocytosis and electrolyte imbalance depending on the level of the patient’s hydration [1].

Imaging is required for the diagnosis of gallstone ileus. In plain abdominal X-ray, Rigler et al. identified four typical landmarks for gallstone ileus: intestinal obstruction (partial or complete), aerobilia, aberrantly located gallstone, and position alterations of the demonstrated gallstone [8]. The evidence of two out of the first three signs is specific for gallstone ileus and represents 40% of total cases [8,9]. Abdominal ultrasonography might help the diagnosis because of its greater ability to detect aerobilia and extra-biliary gallstones. In combination with abdominal X-ray, sonography improves the diagnosis sensitivity into 74% [10]. CT-scan is superior to X-ray and sonography in diagnosing gallstone ileus with a sensitivity of 93% [11]. Rigler’s triad is easily detected with CT-scan compared to X-ray and sonography (77.8%, 14.8%, and 11%, respectively) [11]. Sensitivity, specificity, and accuracy of gallstone ileus diagnosis using CT-scan ranges around 95–100% [11]. In addition, CT-scan with gastrographin contrast is able to detect a fistula, impacted stones, as well as aerobilia [11]. However, in a low-resource setting, abdominal CT-scan with or without contrast is often not available, especially in the emergency room. Thorough anamnesis and clinical examination should consider gallstones as a potential cause of mechanical gastrointestinal obstruction in patients with biliary-related risk factors including middle age women and overweight patients.

A gallstone with a diameter of more than 2.5 cm could induce intestinal obstruction because smaller one generally passes through the intestine spontaneously [1]. Gallstones enter the gastrointestinal system through the cholecysto-enteric fistula, and the most common is the cholecysto-duodenal type [1]. The impaction is predominantly in the distal-end of ileum and the ileocecal valve as the smallest size of the intestine in which the peristaltic force is also weaker than the other parts of the intestines [12].

Treatment of bowel obstruction due to gallstones usually involves an emergency exploratory laparotomy to remove the gallstones and to inspect biliary-intestinal fistula for evacuating of the remaining stones [1]. The recommended surgical procedure for gallstone ileus is still an issue of ongoing debate with several options including enterotomy alone, enterotomy with cholecystectomy and fistula closure, bowel resection alone, and bowel resection with fistula repair [1,3]. In addition, enterotomy followed by fistula repair might be performed in one or two-stage surgery. Enterotomy alone remains the most preferred procedure given the incidence of recurrence and complications are relatively low [5,13]. Removal of the gallbladder is sometimes performed to prevent recurrence, cholangitis, and malignancy arising from the gallbladder which occurs in 5% of patients undergoing enterolithotomy alone [4]. More than 50% cases of gallstone ileus show spontaneous closure of the cholecysto-enteral fistula [1,13]. However, the most important step during emergency laparotomy is the careful assessment of the entire bowel, gallbladder, and post-hepatic biliary systems to remove additional gallstones and to exclude the presence of bile leakage, active inflammation, or necrosis [1,13]. Although cholecystectomy and fistula repair might reduce recurrence and additional biliary complications, the procedure is particularly recommended for selected patients with stable hemodynamic and good general condition [1,13]. In the emergency setting, cholecystectomy and fistula repair are time-consuming and technically challenging causing a higher risk of poor outcome especially in elderly patients with pre-existence of comorbidity [1,3,13]. Enterolithotomy alone is considered the best practice as indicated by a study involving more than 3000 cases in which mortality rates were 5% compared to 7.25% in enterolithotomy with cholecystectomy and fistula repair [13].

Although an infrequent case, gallstone impaction should be considered as the potential cause of small bowel mechanical obstruction in the emergency room especially in patients with risk factors including middle-age women, and patients who are overweight/obese, with or without biliary symptoms. Because sophisticated imaging is not always available in the case of emergency presentation, early exploratory laparotomy should be performed without delay because of excessive time waiting for the imaging.

## Sources of funding

We report no involvement of any sponsor or funding body for this study.

## Ethics approval

Ethical approval is not required at our Institution for case reports.

## Consent

Written informed consent for this case report and any accompanying images was obtained from the patient. A copy of the written informed consent is available upon request. Patient identifying related material was not used in this manuscript.

## Authors’ contribution

SLA conceptualized the first draft and finalized the manuscript. WSA and LC gave expertise in the imaging and reviewed the manuscript. SLA and HPH were involved in the surgery and care of the patient. All authors read and approved the final manuscript.

## Registration of research study

Not applicable for case reports.

## Guarantor

Sumadi Lukman Anwar.

## Provenance and peer review

Not commissioned, externally peer reviewed.

## Declaration of Competing Interest

All authors have declared that they have no potential competing interests.
